# Induction of Labor After Fetal Demise in Third Trimester—A Retrospective Cohort Study

**DOI:** 10.3390/clinpract15110210

**Published:** 2025-11-17

**Authors:** Sara Vodopivec, Gorazd Kavšek, Polona Pečlin, Mirjam Druškovič

**Affiliations:** 1 Faculty of Medicine, University of Ljubljana, 1000 Ljubljana, Slovenia; 2 Department of Perinatology, Division of Obstetrics and Gynecology, University Medical Centre Ljubljana, 1000 Ljubljana, Slovenia

**Keywords:** misoprostol, dinoprostone, fetal demise, stillbirth, termination of pregnancy beyond 28 weeks, labor induction

## Abstract

**Objectives**: The aim of our study was to assess the efficacy and safety of two different labor induction methods in patients after fetal demise beyond 28 weeks, with an unfavorable cervix: misoprostol—prostaglandin E1 analog (PGE1) and dinoprostone—prostaglandin E2 analog (PGE2). **Methods**: This retrospective single-center cohort study included all labor cases after fetal demise (intrauterine fetal death or termination of pregnancy with feticide) from 28 to 40 weeks of gestation, where labor was induced by either PGE1 or PGE2. The primary outcome was the induction-to-delivery time interval. Secondary outcomes included the proportion of patients who delivered within 24 h, the failed induction rate, the length of labor, pain during induction, the adverse outcome rate, and the post-labor hospital stay. **Results**: The induction-to-delivery time interval was shorter in the PGE1 group (*p* = 0.048). There was no statistically significant difference in the proportion of patients who delivered within 24 h (*p* = 0.651) and failed inductions (*p* = 0.18) between groups. The duration of labor was longer in the PGE2 group (*p* = 0.01). Oxytocin augmentation was more common in the PGE2 group (*p* < 0.001). Pain during induction was greater in women in the PGE1 group (*p* < 0.001). There were no statistically significant differences in adverse effects between groups. There was no significant difference in induction to delivery interval between the two methods when comparing lower and higher gestational ages (28 to 34 weeks, *p* = 0.18; 35 to 40 weeks, *p* = 0.343). **Conclusions**: Our findings support the use of a PGE1 regimen for third-trimester labor induction after fetal demise, when no contraindications exist. This approach appears to improve the efficiency of induction and may enhance overall patient care by reducing intervention needs.

## 1. Introduction

Stillbirth is defined as an intrauterine fetal death after the 22nd week of pregnancy or when the fetus weighs 500 g or more [1]. It can occur spontaneously due to abnormal placentation, fetal growth abnormalities, umbilical cord pathology, fetal genetic and structural abnormalities, infection, maternal medical conditions, and intrapartum reasons—intrauterine fetal death (IUFD). Stillbirth can also occur following termination of pregnancy (TOP) for medical reasons [2]. TOP in the third trimester is performed by feticide with intracardiac potassium chloride administration, followed by labor induction, and is predominantly requested due to late-detected fetal abnormalities.

In Slovenia, TOP is legal and can be performed at a woman’s request until the 10th week of pregnancy. After that (with no upper gestational age limit legally defined), special authorization is required, granted by a commission comprising a specialist in obstetrics and gynecology, a specialist in internal or family medicine, and a social worker or psychologist. This commission assesses whether the request for TOP is medically justified. These are predominantly cases of late-detected fetal anomalies [3].

In Slovenia, approximately 100 stillbirths occur annually (98 in 2021), comprising IUFDs and TOPs for medical reasons [1]. Most are managed at the Department of Perinatology, University Medical Centre Ljubljana, which is the largest maternity unit in Slovenia. In 2021, we managed nine stillbirths and 21 TOPs at our institution. With liberal abortion legislation, our institution also provides late TOP services for medical reasons to patients from neighboring countries, where late TOP is either prohibited or inaccessible due to widespread conscientious objection [4]. IUFDs and third-trimester TOPs represent less than 1% of all labors at our institution. However, each case is exceedingly stressful and psychologically devastating for the patient, their family, and the healthcare team. Alleviating this experience is a priority, and our aim is to provide effective induction and labor with minimal pain and adverse effects.

Available studies comparing induction methods after IUFD or TOP in the third trimester are insufficient, and the ideal induction method for third-trimester stillbirth remains undetermined internationally [5]. There are very few studies comparing different methods of labor induction after 28 weeks of gestation in IUFD or after TOP. So far, it has been shown that Foley catheter induction has better efficacy and acceptability than prostaglandin E2 induction when the cervix is favorable [6]. In another study, amniotomy followed by oxytocin infusion was most favorable; however, this also encompassed cases with a favorable cervical status [7]. A prospective randomized study comparing misoprostol vaginally and intracervical dinoprostone gel showed that misoprostol was more effective [8]. Moreover, it was shown that it is sensible to use misoprostol in combination with mifepristone after intrauterine fetal death since it shortens the induction-to-delivery time significantly [9,10].

The mifepristone–misoprostol regimen is recommended by the WHO [11]. At our institution, mifepristone–misoprostol has been the most commonly used method for TOPs following feticide since 2007, while dinoprostone is more frequently used after IUFD [12]. Nonetheless, the physician, based on their personal experience, makes the choice of induction method, and these methods have been used interchangeably.

Noticing the scarcity of the literature in the field and lacking relevant scientific data, we conducted a retrospective single-center cohort study over five years in order to compare the effectiveness, acceptability, and safety of two protocols for inducing labor with a non-viable fetus and an unfavorable cervical status in the third trimester. Our aim was to identify a superior induction method for these cases.

## 2. Materials and Methods

### 2.1. Subjects and Study Design

A retrospective cohort study included all pregnant women who gave birth at the Department of Perinatology, Division of Gynecology, University Medical Centre Ljubljana, between 1 January 2017 and 31 December 2021, where either IUFD or TOP occurred from 28 to 40 weeks of gestation, and labor was induced due to an unfavorable cervix, defined as a Bishop Score of ≤6.

Women were categorized into two study groups according to the method of induction:

- PGE1 Group—Mifepristone–misoprostol (prostaglandin E1 analogue). At our institution, the induction protocol for third-trimester pregnancies consists of 200 mcg of mifepristone orally, followed by hospitalization and misoprostol administration 36–48 h later,200 μg vaginally, followed by 100 μg bucally every 3 h, for 12 h. The daily maximum dosage is 600 μg. If no labor emerges, misoprostol is repeated after 12 h at a higher dose: 200 μg administered vaginally, followed by 200 μg buccally every 3 h for 12 h. If the second cycle is unsuccessful and labor has not begun after 48 h, it is considered a failed induction, and the institutional board determines the next steps. Available options include Foley catheter induction or intra-amniotic or intramuscular administration of the prostaglandin F2 analogue carboprost. In cases of TOP, mifepristone is started simultaneously with the feticide.
- PGE2 Group—Dinoprostone (prostaglandin E2 analogue) vaginally, either in the form of Prostin E2 3 mg vaginal tablets or Propess 10 mg vaginal delivery system.

A Prostin E2 3 mg tablet is inserted into the posterior fornix and repeated after 6 h; the maximal dosage is 6 mg (2 tablets) in 24 h.

Propess 10 mg is placed in the posterior fornix for up to 24 h. If no labor emerges, the dosing is repeated after 24 h from the first administration. If the second cycle is unsuccessful, and labor has not commenced after 48 h, the induction is considered failed; therefore, we proceed with Foley catheter induction.

Exclusion criteria were gestational age less than 28 weeks, premature rupture of membranes, spontaneous labor without induction, Bishop Score > 6 at admission, twin pregnancy with only one non-viable fetus, and contraindication for vaginal delivery.

In active labor, parturients were admitted to the labor room and managed following standard protocols. If the Bishop score was >6 and active labor had not occurred, women were transferred to the labor room for artificial rupture of membranes. Labor analgesia was offered and introduced, and augmentation of labor with oxytocin was used, where appropriate.

Patient data were extracted from computerized medical records and from the Slovenian National Perinatal Information System (NPIS). The NPIS is a population registry that registers all births at ≥22 weeks’ gestation or birth weight ≥ 500 g. Registration is mandatory by law, and more than 140 variables are entered immediately postpartum into a computerized database, which is regularly validated for accuracy.

The primary outcome was an induction-to-delivery time interval that indicates the time from the first dose of prostaglandin to expulsion of the fetus. Secondary outcomes included the proportion of patients who delivered within 24 h, failed induction rate, adverse outcome rate during induction and labor (at least one of the following: nausea, vomiting, chills, fever in labor > 38.5 °C, measured axillary, intrapartum or postpartum bleeding >500 mL, a need for blood transfusion, and obstetric anal sphincter injury), pain during induction (maximum VAS score before transfer to delivery), and post-labor hospital stay.

### 2.2. Statistical Analysis

Patients were categorized into two groups by induction method. Moreover, we opted to analyze certain parameters by stratification of the patients into two groups regarding the gestational age (GA), with GA up to 34 weeks and GA at or over 35 weeks. The two groups were compared for statistically significant differences using the chi-square test for categorical variables and the t-test or Mann–Whitney test for numerical ones. A *p*-value lower than 0.05 was considered statistically significant. IBM SPSS Statistics v29 was used for the analysis.

## 3. Results

During the five-year study period, 115 women met the inclusion criteria, 98 in the PGE1 group and 17 in the PGE2 group. Mean maternal age was 31 ± 6.1 years; mean gestational age was 32 ± 3.4 weeks. In total, 51.3% of primiparous women and 94.8% of all women had no previous caesarean section. All multiparous participants, regardless of prior cesarean delivery, were included in the analysis. Mean birthweight was 1829 ± 748 g. There were 84 cases of TOP (73%) and 31 cases of IUFD (27%) in our study group.

The demographic and obstetric characteristics of both groups are shown in Table 1.

All deliveries were vaginal. Induction and labor outcomes are outlined in Table 2.

A significantly higher proportion of women in the PGE1 group received analgesia during induction compared to the PGE2 group (92.8% vs. 11.8%, *p* < 0.001), as shown in Table 3. Furthermore, analgesic treatment during induction was more frequently needed at lower gestational ages compared to higher gestational ages (94% vs. 45.2%, *p* < 0.001).

The induction-to-delivery interval was not correlated to the reason for stillbirth (1097 ± 970 min for the feticide group; 1240 ± 960 min for the IUFD group, *p* = 0.48).

**Figure 1:**
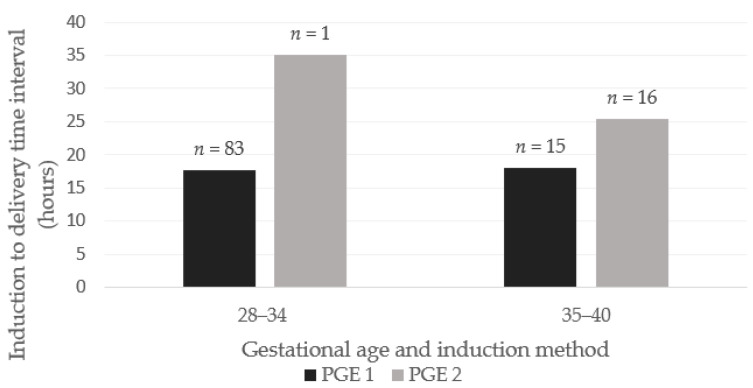
Induction-to-delivery time interval for PGE1 and PGE2 groups, stratified by gestational age. PGE1—misoprostol, PGE2—dinoprostone.

There was no significant difference in induction-to-delivery interval between the two methods when comparing lower and higher gestational ages (28 to 34 weeks, *p* = 0.18; for 35–40 weeks, *p* = 0.343), as shown in Figure 1.

Table 4 shows the results of the comparison by gestational age.

There was no statistically significant difference in adverse effects between the groups. In the PGE1 group, there was one case of diarrhea, five cases of nausea during induction, three cases of vomiting, and two cases of chills. No cases of fever > 38.5 °C were observed during induction in either group. Intrapartum analgesia use was similar among groups, *p* > 0.05 (Table 5).

The distinction between analgesic treatment during induction (Table 3) and intrapartum analgesia (Table 5) was based on the onset of the active phase of labor. Analgesic treatments administered before the establishment of regular uterine contractions and/or cervical dilation of 4–6 cm were classified as given during induction, whereas treatments administered after this point were categorized as intrapartum analgesia. In addition, these two processes are spatially separated: labor induction takes place in the obstetric ward, and once the criteria for the active phase of labor are met (regular contractions and cervical dilation of at least 4 cm), women are transferred to the delivery room.

There were five cases (5.1%) of postpartum hemorrhage over 500 mL in the PGE1 group compared to none in the PGE2 group.

As shown in Table 2, there were three failed inductions (3%) in the PGE1 group. The first was managed with a single intra-amniotic injection of carboprost (1 mg), the second with intramuscular injections of carboprost (0.25 mg every 3 h, for a total of four doses), and the third with a Foley catheter intracervically combined with intramuscular injections of carboprost (0.25 mg every 3 h, for a total of four doses). There was one failed induction in the PGE2 group (5.9%), further managed with Foley catheter induction.

There was one case of uterine rupture in the PGE1 group, which occurred during labor following intrauterine fetal death at 33 weeks of gestation in a patient with no previous uterine scar but with one prior vaginal delivery. Labor induction was initiated with 200 µg of vaginal PGE1, followed by two additional doses of 100 µg administered buccally. Regular contractions began two hours after the final PGE1 dose. The patient received epidural analgesia, and oxytocin augmentation (30 mL/h) was started at 9 cm cervical dilation due to inadequate contractions. Full dilation was achieved 6 h after the last PGE1 dose, and the second stage of labor lasted less than 30 min. The fetus weighed 2090 g. No injuries were detected immediately after delivery, and vaginal bleeding was within normal limits. On the following day, the patient developed lower abdominal pain and urinary retention. Ultrasound revealed an 8 cm mixed echogenic mass communicating with the cervical canal, consistent with a parametrial hematoma, and a cervical tear was seen. Vaginal examination revealed a 5 cm vaginal tear in the left fornix extending to the lateral cervix; the apex of the tear was neither visible nor palpable, and the laceration was sutured under general anesthesia. Six days later, the patient developed symptoms and signs of infection and elevated inflammatory markers. Imaging showed features of an infected parametrial hematoma. Diagnostic laparoscopy, converted to laparotomy, revealed a lower uterine segment rupture extending from the cervical tear. The hematoma was evacuated, the tear was sutured, and the patient recovered well. She subsequently delivered a healthy child three years later.

No anal sphincter injuries were observed. One patient in the PGE1 group required a blood transfusion after labor, and there was one case of late postpartum bleeding in the PGE1 group.

Regarding late complications, we only had data for 34 patients. The complications assessed did not differ significantly between groups:

– Uterine curettage up to three months after labor: one case in the PGE1 group and one in the PGE2 group;
– Hysteroscopy for retained products of conception: one case in the PGE2 group;
– Endometritis and antibiotic treatment after labor: three cases in the PGE1 group and one case in the PGE2 group;
– Late postpartum bleeding: one case in the PGE1 group, treated with US-guided curettage.

## 4. Discussion

In the present study, we aimed to assess the efficacy and safety of two different prostaglandins for labor induction in the third trimester in circumstances of an unfavorable cervix and non-viable fetuses. Our study shows that the mifepristone–misoprostol (PGE1) induction method is more efficient in terms of a shorter induction-to-delivery interval compared to vaginal dinoprostone (PGE2). Both methods have similar rates of delivery in 24 h, as well as failed induction rates. Labor augmentation with oxytocin was more frequent, and the duration of labor was longer in the PGE2 group. The adverse effects rates were low and comparable between the two groups.

As seen from the size of the two groups, the use of the PGE1 protocol prevails over PGE2 induction for third-trimester stillbirths at our institution, which is in accordance with WHO recommendations [11]. As also observed in previous studies by Biswas et al. and De Heus et al., PGE1 has been shown to be more effective than PGE2 [8,13]. The main advantages of PGE1 include its effectiveness, lower cost, stability at room temperature, ease of administration, and reduced need for oxytocin [14,15]. In regard to our results, the advantages are also a shorter hospital stay, less postpartum hemorrhage, and a reduced need for oxytocin augmentation. Conversely, one study reported a shorter induction-to-delivery time for PGE2 compared to PGE1 in second- and third-trimester IUFD; however, this study involved significant variation in PGE1 dosing [16]. While oral PGE1 is increasingly used for labor induction at term, we believe that vaginal PGE1 remains a more effective protocol for managing stillbirth [17,18].

While previous studies have reported greater pain with PGE2 induction, we observed higher pain scores and increased use of analgesics in the PGE1 group [8,13]. This may reflect the strong gestational age imbalance between groups, as earlier gestations, smaller fetal size, and less favorable cervical status can contribute to inductions that are more painful. Thus, the differences in pain and analgesic requirements may not be attributable to the induction agent itself. While stratified analyses provide some context, residual confounding cannot be excluded, and these findings should be interpreted cautiously.

Having two discordant groups with significantly lower gestational ages and newborn birthweight in the PGE1 group probably had an impact on the outcomes. This is why we decided to also perform an analysis by gestational age. That showed that PGE1 was more effective even in higher gestational ages compared to PGE2. Therefore, we conclude that it would be appropriate to use misoprostol induction even in fetal demise in the late third trimester, contrary to our current clinical practice.

We seldom use the balloon catheter as a method for labor induction, although it has previously been shown to be effective, with a shorter induction-to-delivery interval compared to PGE1 and PGE2 induction [6]. Previous research from our department also indicated that pain with balloon catheter induction was lower compared to PGE2 induction, while induction-to-delivery time intervals were comparable [19]. It is important to acknowledge the risk of uterine rupture in a previously scarred uterus, which is higher with prostaglandins but negligible with balloon catheter use [20,21]. Therefore, it would be sensible to systematically consider balloon catheter induction in cases with a scarred uterus, particularly at higher gestational ages [7,22]. The occurrence of uterine rupture in a primigravid patient with no previous uterine scar, while rare, highlights the potential risks of misoprostol use at higher gestational ages even in unscarred uteri. Misoprostol is a potent uterotonic, and uterine sensitivity may increase with advancing gestation, potentially elevating the risk of hyperstimulation or rupture [11]. This underscores the need for careful dosing, close monitoring of uterine activity, and individualized risk assessment when using misoprostol for induction in the late third trimester. Although such events are uncommon, clinicians should remain vigilant and counsel patients accordingly. A recent randomized controlled trial demonstrated it might be sensible to use cervical sensitizers (a combination of both mifepristone and Foley catheters) in order to lower the required dose of oxytocin for augmentation [23]

In the context of a deceased fetus, it is crucial to consider the emotionally devastating situation for the woman. For IUFD, it has been shown that a longer diagnosis-to-labor interval increases anxiety in these patients [24]. Considering this, initiating induction with mifepristone immediately after the diagnosis of IUFD could have a positive psychological impact. It may reassure the woman that she is being cared for and that treatment has started promptly to end the overwhelming situation she is facing. Shared decision-making is a cornerstone of respectful obstetric care. Therefore, it is essential to consider women’s wishes and expectations regarding delivery, especially in fragile patients after fetal demise who may experience unfruitful labor. Among these patients, the option of expectant management should also be considered [25]. Moreover, it is vital to provide sufficient analgesic treatment. According to our results and the fact that pain is significantly higher in lower gestational ages, we will consider epidural analgesia from the beginning of induction for early-third-trimester stillbirths in the future. Studies have shown that acceptance and satisfaction with the induction process are related to the duration of induction [26].

Our study has several limitations. A key limitation is the unequal group sizes, with 98 participants in the PGE1 group and 17 in the PGE2 group, as well as significant baseline differences in gestational age and birth weight (Table 1). These factors are a consequence of the fact that our study group sizes were determined by the study period, including all consecutive eligible cases over five years, and they could have influenced outcomes, such as induction-to-delivery interval, pain intensity, and oxytocin use. Due to the retrospective design and the small size of the PGE2 group, multivariable or propensity score analyses were not feasible. To mitigate this, we conducted stratified analyses by gestational age and reported baseline characteristics transparently, allowing readers to assess potential confounding. While residual confounding cannot be entirely excluded, our findings indeed provide valuable insights into labor induction outcomes with PGE1 and PGE2 in a real-world clinical setting.

This study was not sufficiently powered to detect differences in rare outcomes, such as serious adverse events. A post hoc power analysis indicated only 40.3%, suggesting that the sample size was insufficient to detect smaller or rare differences between groups, including serious adverse events, increasing the risk of Type II errors and limiting the ability to draw firm conclusions regarding safety outcomes. Extending the study period could increase the sample size, particularly for the smaller PGE2 group, and improve power for detecting both common and rare outcomes. However, this may introduce variability due to changes in clinical practice over time and would require careful consideration of data consistency and quality.

The choice of induction method was left to the attending physician, potentially introducing selection bias—for instance, dinoprostone may have been preferentially used in more advanced gestations or in cases of intrauterine fetal death. This physician-driven allocation could have influenced outcomes such as induction-to-delivery interval, analgesic requirements, and oxytocin use. Although baseline characteristics were reported and stratified analyses performed, readers should interpret the results with this limitation in mind.

Patient-centered outcomes, such as satisfaction, emotional impact, and analgesia adequacy, which are particularly relevant in the context of delayed induction, were not assessed, which is inherent to the retrospective design. Although TOP and IUFD have different pathophysiologic mechanisms, labor induction in TOP cases is, at our institution, always initiated at least one day after fetal demise (feticide), similar to what happens in IUFD. As such, induction management was likely comparable between TOP and IUFD cases in our cohort. Lastly, follow-up data were unavailable for the majority of the patients, as postnatal care and follow-up in Slovenia are typically performed by primary care gynecologists or the relevant home institution for foreign patients.

On the other hand, to our knowledge, this is the first study directly comparing two medication methods, misoprostol and dinoprostone, for labor induction in the third trimester in cases of fetal demise with an unfavorable cervix. We present one of the largest cohorts on this topic in the current literature, with detailed clinical data from our 5-year work in the field. This study reflects standardized clinical protocols and consistent management practices in our institution, enhancing the reliability of the observation. Furthermore, it serves as a comprehensive audit of our clinical experience and provides practical insights for patient counseling and clinical decision-making. Our results contribute to the evidence on the efficacy and safety of third-trimester pregnancy termination. Given the emotional and clinical challenges associated with stillbirth management, particularly with an unfavorable cervix and expected latency of labor, our data are highly relevant for clinicians internationally. Finally, this study lays a foundation for future prospective research to confirm and expand upon these findings, supporting both efficacy and safety evaluations of third-trimester induction regimens.

## 5. Conclusions

Based on our results and experience, we conclude that it would be sensible to use the mifepristone–misoprostol regimen for all inductions of labor after fetal demise in the third trimester when there is no contraindication to the medication due to its shorter induction-to-delivery time interval, shorter duration of labor, reduced oxytocin use during labor, and more convenient application. However, it is of paramount importance, especially in the case of a deceased fetus, to properly assess and address pain during induction and provide sufficient analgesia throughout both the induction and labor processes. The limitations stated above highlight the need for further prospective studies with a broader range of observed outcomes and a larger sample size, sufficiently powered to evaluate both clinical and patient-reported outcomes, in order to better understand the overall impact of induction method on effectiveness, safety, and patient satisfaction, to confirm our retrospective findings.

## Figures and Tables

**Table 1:** Maternal demographic and obstetric data with respect to different induction methods.

	PGE1 Group *n* = 98	PGE2 Group *n* = 17	*p*-Value
Maternal age (years)	31.2 ± 6.1	30.5 ± 5.5	0.65
Gestational age (weeks)	31.4 ± 2.7	37.5 ± 1.7	<0.001
BMI	23.7 ± 4.9	23.5 ± 4.5	0.9
Primiparous	51 (52%)	8 (47.1%)	0.7
Multiparous	47 (48%)	9 (52.9%)	0.7
Multiparous with previous caesarean delivery	6 (6. 1%)	0	0.29
Newborn birthweight (grams)	1666 ± 626	2783 ± 710	<0.001
Reason for stillbirth IUFD TOP	14 (14.3%) 84 (85.7%)	17 (100%) 0	NA

Data are presented as mean (SD) or *n* (%). PGE1: prostaglandin 1, PGE2: prostaglandin 2, BMI: body mass index, IUFD: intrauterine fetal death, TOP: termination of pregnancy, NA: not applicable.

**Table 2:** Induction and labor outcomes.

	PGE1 Group *n* = 98	PGE2 Group *n* = 17	*p*-Value
Induction to delivery interval—mean ± SD (hours)	17.7 ± 15.2	26.1 ± 19.2	0.048
Delivery in 24 h—*n* (%)	82 (83.6)	13 (76.5)	0.651
Failure to induce—*n* (%)	3 (3.1)	1 (5.9)	0.18
Duration of labor—mean ± SD (hours)	2.85 ± 1.79	4.44 ± 2.57	0.01
Pain during induction (VAS max)	4.0 ± 1.9	2.1 ± 0.8	<0.001
Analgesic treatment received during induction—*n* (%)	91 (92.9)	3 (17.6)	<0.001
Oxytocin augmentation of labor—*n* (%)	23 (23.5)	12 (70.6)	<0.001
Post-labor hospital stay—mean ± SD (hours)	24.1 ± 16.4	33.6 ± 48.8	0.14

Data are presented as mean (SD) or *n* (%) and with a significance threshold at *p* < 0.05. PGE1: prostaglandin 1, PGE2: prostaglandin 2, VAS max: maximum visual analogue score before transfer to the delivery room.

**Table 3:** Analgesic treatment during induction.

	PGE1 Group *n* = 98	PGE2 Group *n* = 17
None	7 (7.1)	14 (82.4)
Weak (NSAID, paracetamol)	76 (78.4)	2 (11.8)
Strong (NSAID/paracetamol + opiate)	14 (14.4)	1 (5.9)

Data are presented as *n* (%). PGE1: prostaglandin 1, PGE2: prostaglandin 2, NSAID: non-steroidal anti-inflammatory drugs.

**Table 4:** Analysis by gestational age.

	GA 28–34 Weeks (*n* = 84)	GA 35–40 Weeks (*n* = 31)	*p*-Value
PGE1 as induction method	83 (98.8)	15 (48.4)	NA
PGE2 as induction method	1 (1.2)	16 (51.6)	NA
Induction to delivery time interval—mean ± SD (hours)	17.7 ± 15.8	20.6 ± 15.4	0.274
Pain during induction (VAS max)—mean ± SD	4.1 ± 1.9	2.7 ± 1.3	<0.001
Analgesic treatment received during induction—*n* (%)	78 (94)	14 (45.2)	<0.001
Oxytocin augmentation of labor—*n* (%)	18 (21.4)	17 (54.8)	<0.001
		PGE1: 5 (33) PGE2: 12 (75)	0.02
Duration of labor—mean ± SD (hours)	2.8 ± 1.7	3.9 ± 2.5	0.038

Data are presented as mean ± standard deviation (SD) or *n* (%) and with a significance threshold at *p* < 0.05. PGE1: prostaglandin 1; PGE2: prostaglandin 2; GA—gestational age; VAS max: maximum visual analogue score before transfer to the delivery room; NA—not applicable.

**Table 5:** Intrapartum analgesia.

	PGE1 Group *n* = 98	PGE2 Group *n* = 17
No or inhalation analgesia (N_2_O)	24 (24.5)	3 (17.6)
Pethidine or remifentanil i.v.	61 (62.2)	10 (58.8)
Epidural analgesia	13 (13.3)	4 (23.5)

Data are presented as *n* (%). PGE1: prostaglandin 1, PGE2: prostaglandin 2, N_2_O: nitrous oxide.

## Data Availability

The raw data supporting the conclusions of this article will be made available by the authors upon request.

## Abbreviations

The following abbreviations are used in this manuscript:

IUFD	Intrauterine fetal death
TOP	Termination of pregnancy
PGE1	Prostaglandin E1—misoprostol
PGE2	Prostaglandin E2—dinoprostone
VAS	Visual analogue scale

## References

[1] Health Statistical Yearbook of Slovenija 2021. https://nijz.si/publikacije/zdravstveni-statisticni-letopis-2021/.

[2] Silver R.M., Reddy U. (2024). Stillbirth: We can do better. Am. J. Obs. Gynecol..

[3] Pinter B., Aubeny E., Bartfai G., Loeber O., Ozalp S., Webb A. (2005). Accessibility and availability of abortion in six European countries. Eur. J. Contracept. Reprod. Health Care.

[4] Caruso E. (2023). The hyper-regulation of abortion care in Italy. Int. J. Gynaecol. Obstet..

[5] American College of Obstetricians and Gynecologists, Society for Maternal-Fetal Medicine (2020). Management of Stillbirth: Obstetric Care Consensus No, 10. Obstet. Gynecol..

[6] Attali E., Kern G., Fouks Y., Reicher L., Many A., Levin I., Yogev Y., Cohen A. (2022). Labor induction in third trimester non-viable fetus. J. Matern.-Fetal Neonatal Med..

[7] Gawron L.M., Kiley J.W. (2013). Labor induction outcomes in third-trimester stillbirths. Int. J. Gynaecol. Obstet..

[8] Biswas T. (2017). Misoprostol (PGE1) versus dinorostone gel (PGE”) in induction of labour in late intra uterine fetal death with unfavourable cervix: A prospective comparative study. Int. J. Reprod. Contracept. Obstet. Gynecol..

[9] Panda S., Jha V., Singh S. (2013). Role of Combination OF Mifepristone and Misoprostol Verses Misoprostol alone in Induction of Labour in Late Intrauterine Fetal Death: A Prospective Study. J. Fam. Reprod. Health.

[10] Chaudhuri P., Datta S. (2015). Mifepristone and misoprostol compared with misoprostol alone for induction of labor in intrauterine fetal death: A randomized trial. J. Obstet. Gynaecol. Res..

[11] WHO (2011). Recommendations for Induction of Labour.

[12] Vrhkar N., Šajina S.B., Tul N. (2012). Termination of pregnancy with mifepristone and misoprostol after the 11th week of pregnancy. Zdr. Vestn..

[13] De Heus R., Graziosi G.C., Christiaens G.C., Bruinse H.W., Mol B.W. (2004). Medical management for termination of second and third trimester pregnancies: A comparison of strategies. Eur. J. Obstet. Gynecol. Reprod. Biol..

[14] Wang L., Zheng J., Wang W., Fu J., Hou L. (2016). Efficacy and safety of misoprostol compared with the dinoprostone for labor induction at term: A meta-analysis. J. Matern. Fetal Neonatal Med..

[15] Dodd J.M., Crowther C.A. (2006). Misoprostol versus cervagem for the induction of labour to terminate pregnancy in the second and third trimester: A systematic review. Eur. J. Obstet. Gynecol. Reprod. Biol..

[16] Amin K.V., Chauhan A.R., Goel A. (2019). Current Practices of Cervical Ripening and Induction of Labour in Intrauterine Foetal Demise: An Observational Study. J. Obstet. Gynaecol. India.

[17] Redling K., Schaedelin S., Huhn E.A., Hoesli I. (2019). Efficacy and safety of misoprostol vaginal insert vs. oral misoprostol for induction of labor. J. Perinat. Med..

[18] Dickinson J.E., Evans S.F. (2003). A comparison of oral misoprostol with vaginal misoprostol administration in second-trimester pregnancy termination for fetal abnormality. Obstet. Gynecol..

[19] Pečlin P., Druškovič M., Verdenik I., Kavšek G. (2015). Mechanical vs. pharmacological methods for labour induction: Impact on pain perception and satisfaction. Abstract Book: ECIC 2015.

[20] Aslan H., Unlu E., Agar M., Ceylan Y. (2004). Uterine rupture associated with misoprostol labor induction in women with previous cesarean delivery. Eur. J. Obstet. Gynecol. Reprod. Biol..

[21] Bujold E., Blackwell S.C., Gauthier R.J. (2004). Cervical ripening with transcervical foley catheter and the risk of uterine rupture. Obstet. Gynecol..

[22] Chakhtoura N.A., Reddy U.M. (2015). Management of stillbirth delivery. Semin. Perinatol..

[23] Dasgupta S., Dasgupta J., Goswami B., Mondal J. (2023). Randomized controlled trial comparing efficacy of a combination regime containing two cervical sensitizers (mifepristone + Foley’s catheter) versus single agent mifepristone or Foley’s catheter for labor induction in women attempting TOLAC at late third trimester with a dead fetus in utero. J. Obstet. Gynaecol. Res..

[24] Rådestad I., Steineck G., Nordin C., Sjögren B. (1996). Psychological complications after stillbirth--influence of memories and immediate management: Population based study. BMJ.

[25] Silver R.M., Heuser C.C. (2010). Stillbirth workup and delivery management. Clin. Obstet. Gynecol..

[26] Akoury H.A., Hannah M.E., Chitayat D., Thomas M., Winsor E., Ferris L.E., Einarson T.R., Seaward P., Ryan G., Willan A.R. (2004). Randomized controlled trial of misoprostol for second-trimester pregnancy termination associated with fetal malformation. Am. J. Obstet. Gynecol..

